# *catena*-Poly[[[(2,2′-bipyridine-κ^2^ *N*,*N*′)cobalt(II)]-μ-(*E*)-3,3′-(but-2-ene-2,3-di­yl)dibenzoato-κ^4^ *O*,*O*′:*O*′′,*O*′′′] hemihydrate]

**DOI:** 10.1107/S1600536811041699

**Published:** 2011-10-22

**Authors:** Zong-Sheng Li, Seik Weng Ng

**Affiliations:** a College of Safety and Environmental Engineering, Capital University of Economics and Business, Beijing 100070, People’s Republic of China; b Department of Chemistry, University of Malaya, 50603 Kuala Lumpur, Malaysia; c Chemistry Department, King Abdulaziz University, PO Box 80203 Jeddah, Saudi Arabia

## Abstract

The title coordination polymer, {[Co(C_18_H_14_O_4_)(C_10_H_8_N_2_)]·0.5H_2_O}_*n*_, features a helical polymeric chain that runs along the *b* axis. The Co atoms are chelated by the carboxyl­ate groups of two 3,3′-(but-2-ene-2,3-di­yl)dibenzoate ligands and the N atoms of a 2,2′-bipyridine ligand. The lattice water mol­ecule is disordered about a center of inversion and is connected to the chain by an O—H⋯O hydrogen bond. The Co^II^ atom shows a distorted octa­hedral coordination.

## Related literature

For a review of the adducts of metal carboxyl­ates with 2,2′-bipyridine-like ligands, see: Ye *et al.* (2005 ▶). For details of the synthesis, see: McMurry (1989 ▶).
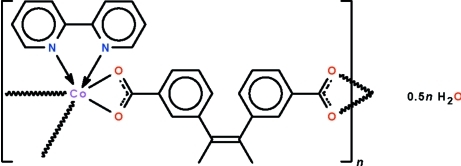


## Experimental

### 

#### Crystal data

[Co(C_18_H_14_O_4_)(C_10_H_8_N_2_)]·0.5H_2_O
*M* *_r_* = 518.41
Monoclinic, 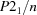

*a* = 8.7028 (9) Å
*b* = 19.872 (2) Å
*c* = 14.5181 (14) Å
β = 97.845 (2)°
*V* = 2487.2 (4) Å^3^
*Z* = 4
Mo *K*α radiation
μ = 0.73 mm^−1^
*T* = 293 K
0.28 × 0.17 × 0.05 mm

#### Data collection

Bruker SMART APEX diffractometer
Absorption correction: multi-scan (*SADABS*; Sheldrick, 1996 ▶) *T*_min_ = 0.822, *T*_max_ = 0.965
14695 measured reflections
5667 independent reflections
3153 reflections with *I* > 2σ(*I*)
*R*_int_ = 0.046

#### Refinement

*R*[*F* ^2^ > 2σ(*F* ^2^)] = 0.045
*wR*(*F* ^2^) = 0.119
*S* = 0.96
5667 reflections
327 parameters
6 restraints
H-atom parameters constrained
Δρ_max_ = 0.23 e Å^−3^
Δρ_min_ = −0.20 e Å^−3^

### 

Data collection: *APEX2* (Bruker, 2005 ▶); cell refinement: *SAINT* (Bruker, 2005 ▶); data reduction: *SAINT*; program(s) used to solve structure: *SHELXS97* (Sheldrick, 2008 ▶); program(s) used to refine structure: *SHELXL97* (Sheldrick, 2008 ▶); molecular graphics: *X-SEED* (Barbour, 2001 ▶); software used to prepare material for publication: *publCIF* (Westrip, 2010 ▶).

## Supplementary Material

Crystal structure: contains datablock(s) global, I. DOI: 10.1107/S1600536811041699/bt5671sup1.cif

Structure factors: contains datablock(s) I. DOI: 10.1107/S1600536811041699/bt5671Isup2.hkl

Additional supplementary materials:  crystallographic information; 3D view; checkCIF report

## Figures and Tables

**Table 1:** Hydrogen-bond geometry (Å, °)

*D*—H⋯*A*	*D*—H	H⋯*A*	*D*⋯*A*	*D*—H⋯*A*
O1w—H11⋯O1	0.84	1.98	2.812 (5)	173

**Fig. 1.**
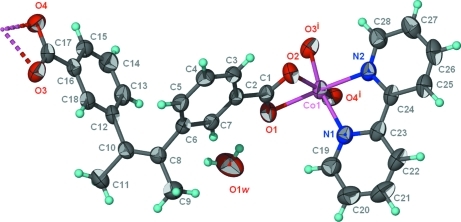
Anisotropic ellipsoid plot (Barbour, 2001) of a portion of polymeric Co(C10H8N2)(C18H14O4).0.5H2O at the 50% probability level; hydrogen atoms are drawn as spheres of arbitrary radius.

**Fig. 2.**
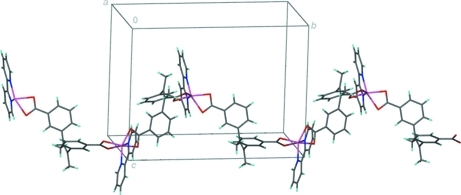
Chain structure; the lattice water molecules are not shown.

## supplementary crystallographic information

### Comment

Meta(II) dicarboxylates generally adopt three-dimensional polymeric architectures as the carboxyl –CO_2_ ends of the dianion are both capable of binding to more than one metal atom. The three-dimensional motifs can be altered to two-dimensional layers or even linear chains through the formation of adducts with 2,2'-bipyridine like ligands; the molecular architectures of such adducts of metal carboxylates with such *α*,*α*'-dimine ligands have been reviewed (Ye *et al.*, 2005).

We have synthesized (*E*)-3,3'-(but-2-ene-2,3-diyl)dibenzoic acid in a multi-step synthesis for use in another research projecttheme; we have used this rigid dicarboxylic acid to form a coordination polymer as no derivatives of this acid have been reported. The dicarboxylate ion of the coordination polymer, [Co(C_10_H_8_N_2_)(C_18_H_14_O_4_)^.^0.5H_2_O]*_n_* (Scheme I, Fig. 1), has its carboxyl –CO_2_ ends each chelating a 2,2'-bipyridine chelated Co^II^ atom to generate a helical polymeric chain that runs along the *b*-axis of the monoclinic unit cell (Fig. 2). The Co^II^ atom shows octahedral coordination. The lattice water molecule is disordered about a center-of-inversion and is connected to the chain by an O–H···O hydrogen bond (Table 1).

### Experimental

2,3-Bis-(3-bromophenyl)-2-butene was synthesized from the cross-coupling of 3-bromoacetophenone catalyzed by low-valent titanium (McMurry, 1989).

2,3-Bis-(3-bromophenyl)-2-butene (17.3 g, 0.048 mol) was added to a solution of *n*-butyllithium (2.5 *M* in hexane, 40 ml, 0.10 mol) in ether (400 ml) at 200 K. The reaction was carried out under nitrogen. The mixture was warmed to room temperature and then stirred overnight. The reaction was quenched with water (100 ml) and the organic compounds were extracted with ether. The aqueous phase was adjusted to a pH of 1 by the addition of concentrated hydrochloric acid. The precipitate was collected, washed with ethyl acetate and dried to yield 3,3'-(but-2-ene-2,3-diyl)dibenzoic acid (yield 9.8 g, 70%) as a mixture of *E* and *Z* isomers.

3,3'-(But-2-ene-2,3-diyl)dibenzoic acid (10 g, 0.034 mol), thionyl chloride (12.0 ml) and methanol (150 ml) were heated for 2 h. The solvent was evaporated and the residue was purified by silica gel chromatography to yield 2.2 g of (*Z*)-dimethyl 3,3'-(but-2-ene-2,3-diyl)dibenzoate and 6.6 g of (*E*)-dimethyl 3,3'-(but-2-ene-2,3-diyl)dibenzoate.

To (*E*)-dimethyl 3,3'-(but-2-ene-2,3-diyl)dibenzoate (5.0 g,15.4 mmol) in THF (30 ml) was added lithium hydroxide (1.48 g, 61.6 mmol) in water (30 ml). The mixture was stirred overnight. The solvent was removed and then acidified with concentrated hydrochloric acid; the reaction was carried out at 273 K. The precipitate was collected, washed with ethyl acetate and dried to give to (*E*)-3,3'-(but-2-ene-2,3-diyl)dibenzoic acid (yield 4.3 g, 95%) as a white solid.

(*E*)-3,3'-(But-2-ene-2,3-diyl)dibenzoic acid (15.3 mg, 0.05 mmol), sodium hydroxide (4.1 mg, 0.10 mmol), cobalt(II) chloride hexahydrate (11.8 mg, 0.05 mmol) and 2,2'-bipyridine (7.8 mg, 0.05 mol) were mixed in water (4 ml). This was transferred to a 25-mLTeflon-lined stainless-steel Parr bomb. The bomb was heated at 408 K for 3 days. The bomb was cooled slowly to room temperature. Red block-shaped crystals were obtained; yield: 10.8 mg (40% based on the acid). CH&N elemental analysis. Calcd. (%): C, 65.00; H, 4.22; N, 5.33. Found (%): C, 65.29; H, 4.05; N, 5.32.

### Refinement

Carbon-bound H-atoms were placed in calculated positions (C—H 0.93 to 0.96 Å) and were included in the refinement in the riding model approximation, with *U*(H) set to 1.2 to 1.5*U*(C).

The water molecule lies near a center-of-inversion, and was assigned half site-occupancy. The H atoms were placed in a chemically sensible position on the basis of one hydrogen bonding interaction.

### Crystal data

**Table:**

[Co(C_18_H_14_O_4_)(C_10_H_8_N_2_)]·0.5H_2_O	*F*(000) = 1072
*M**_r_* = 518.41	*D*_x_ = 1.384 Mg m^−^^3^
Monoclinic, *P*2_1_/*n*	Mo *K*α radiation, λ = 0.71073 Å
Hall symbol: -P 2yn	Cell parameters from 2090 reflections
*a* = 8.7028 (9) Å	θ = 2.6–21.1°
*b* = 19.872 (2) Å	µ = 0.73 mm^−^^1^
*c* = 14.5181 (14) Å	*T* = 293 K
β = 97.845 (2)°	Plate, red
*V* = 2487.2 (4) Å^3^	0.28 × 0.17 × 0.05 mm
*Z* = 4	

### Data collection

**Table:**

Bruker SMART APEX diffractometer	5667 independent reflections
Radiation source: fine-focus sealed tube	3153 reflections with *I* > 2σ(*I*)
graphite	*R*_int_ = 0.046
ω scans	θ_max_ = 27.5°, θ_min_ = 1.8°
Absorption correction: multi-scan (*SADABS*; Sheldrick, 1996)	*h* = −10→11
*T*_min_ = 0.822, *T*_max_ = 0.965	*k* = −23→25
14695 measured reflections	*l* = −18→14

### Refinement

**Table:**

Refinement on *F*^2^	Primary atom site location: structure-invariant direct methods
Least-squares matrix: full	Secondary atom site location: difference Fourier map
*R*[*F*^2^ > 2σ(*F*^2^)] = 0.045	Hydrogen site location: inferred from neighbouring sites
*wR*(*F*^2^) = 0.119	H-atom parameters constrained
*S* = 0.96	*w* = 1/[σ^2^(*F*_o_^2^) + (0.0535*P*)^2^] where *P* = (*F*_o_^2^ + 2*F*_c_^2^)/3
5667 reflections	(Δ/σ)_max_ = 0.001
327 parameters	Δρ_max_ = 0.23 e Å^−^^3^
6 restraints	Δρ_min_ = −0.20 e Å^−^^3^

### Fractional atomic coordinates and isotropic or equivalent isotropic displacement parameters (Å^2^)

**Table:**

	*x*	*y*	*z*	*U*_iso_*/*U*_eq_	Occ. (<1)
Co1	0.72581 (4)	0.433780 (19)	0.65569 (3)	0.05411 (15)	
O1	0.6416 (2)	0.49163 (10)	0.76819 (13)	0.0630 (5)	
O2	0.6521 (2)	0.53304 (10)	0.63038 (14)	0.0648 (6)	
O3	−0.0199 (2)	0.88087 (10)	0.90530 (14)	0.0629 (5)	
O4	−0.2047 (2)	0.83205 (10)	0.81231 (14)	0.0659 (6)	
O1W	0.6236 (8)	0.4682 (3)	0.9574 (3)	0.125 (2)	0.50
H11	0.6219	0.4775	0.9009	0.188*	0.50
H12	0.5499	0.4883	0.9774	0.188*	0.50
N1	0.9576 (3)	0.44197 (11)	0.71738 (18)	0.0564 (6)	
N2	0.8468 (3)	0.41977 (11)	0.54323 (17)	0.0555 (6)	
C1	0.6243 (3)	0.54056 (14)	0.7128 (2)	0.0507 (7)	
C2	0.5758 (3)	0.60753 (14)	0.74361 (19)	0.0468 (6)	
C3	0.5469 (3)	0.66010 (15)	0.68050 (19)	0.0526 (7)	
H3	0.5576	0.6534	0.6183	0.063*	
C4	0.5025 (3)	0.72181 (15)	0.7100 (2)	0.0590 (8)	
H4	0.4817	0.7567	0.6674	0.071*	
C5	0.4887 (3)	0.73232 (15)	0.8025 (2)	0.0571 (7)	
H5	0.4581	0.7744	0.8215	0.069*	
C6	0.5193 (3)	0.68143 (14)	0.86754 (18)	0.0497 (7)	
C7	0.5619 (3)	0.61921 (14)	0.83632 (18)	0.0490 (7)	
H7	0.5817	0.5842	0.8788	0.059*	
C8	0.5183 (3)	0.69319 (14)	0.96955 (19)	0.0547 (7)	
C9	0.6782 (4)	0.69610 (19)	1.0248 (2)	0.0845 (11)	
H9A	0.7295	0.7366	1.0096	0.127*	
H9B	0.7373	0.6577	1.0100	0.127*	
H9C	0.6696	0.6958	1.0900	0.127*	
C10	0.3872 (4)	0.70120 (14)	1.00524 (19)	0.0569 (7)	
C11	0.3799 (4)	0.71311 (19)	1.1077 (2)	0.0856 (11)	
H11A	0.3364	0.6743	1.1338	0.128*	
H11B	0.3160	0.7516	1.1148	0.128*	
H11C	0.4825	0.7210	1.1393	0.128*	
C12	0.2327 (3)	0.70095 (14)	0.94623 (19)	0.0528 (7)	
C13	0.1647 (4)	0.64333 (16)	0.9054 (2)	0.0682 (9)	
H13	0.2170	0.6025	0.9137	0.082*	
C14	0.0202 (4)	0.64560 (16)	0.8527 (2)	0.0714 (9)	
H14	−0.0243	0.6062	0.8268	0.086*	
C15	−0.0583 (3)	0.70517 (15)	0.83819 (19)	0.0570 (7)	
H15	−0.1548	0.7063	0.8017	0.068*	
C16	0.0063 (3)	0.76353 (14)	0.87795 (18)	0.0494 (7)	
C17	−0.0770 (3)	0.82881 (14)	0.8639 (2)	0.0516 (7)	
C18	0.1496 (3)	0.76036 (14)	0.93232 (19)	0.0522 (7)	
H18	0.1916	0.7995	0.9605	0.063*	
C19	1.0053 (4)	0.45294 (16)	0.8071 (3)	0.0727 (9)	
H19	0.9311	0.4587	0.8468	0.087*	
C20	1.1591 (4)	0.45614 (18)	0.8438 (3)	0.0845 (11)	
H20	1.1881	0.4646	0.9068	0.101*	
C21	1.2687 (4)	0.44668 (18)	0.7861 (4)	0.0916 (12)	
H21	1.3736	0.4483	0.8094	0.110*	
C22	1.2228 (4)	0.43482 (16)	0.6937 (3)	0.0752 (10)	
H22	1.2961	0.4282	0.6536	0.090*	
C23	1.0648 (3)	0.43281 (13)	0.6601 (2)	0.0560 (7)	
C24	1.0024 (3)	0.42047 (12)	0.5620 (2)	0.0534 (7)	
C25	1.0942 (4)	0.41078 (15)	0.4925 (3)	0.0668 (9)	
H25	1.2017	0.4130	0.5057	0.080*	
C26	1.0246 (4)	0.39784 (16)	0.4041 (3)	0.0786 (10)	
H26	1.0850	0.3902	0.3570	0.094*	
C27	0.8642 (4)	0.39610 (17)	0.3846 (2)	0.0772 (10)	
H27	0.8151	0.3868	0.3250	0.093*	
C28	0.7805 (4)	0.40859 (16)	0.4565 (2)	0.0690 (9)	
H28	0.6728	0.4093	0.4440	0.083*	

### Atomic displacement parameters (Å^2^)

**Table:**

	*U*^11^	*U*^22^	*U*^33^	*U*^12^	*U*^13^	*U*^23^
Co1	0.0452 (2)	0.0561 (3)	0.0637 (3)	−0.00109 (18)	0.01649 (18)	−0.00717 (19)
O1	0.0733 (14)	0.0552 (13)	0.0637 (13)	0.0107 (10)	0.0214 (10)	−0.0004 (10)
O2	0.0729 (14)	0.0641 (13)	0.0623 (13)	0.0021 (11)	0.0270 (11)	−0.0056 (10)
O3	0.0502 (11)	0.0490 (12)	0.0899 (15)	−0.0008 (9)	0.0108 (10)	−0.0069 (11)
O4	0.0546 (13)	0.0654 (14)	0.0750 (14)	0.0052 (10)	−0.0008 (11)	−0.0053 (11)
O1W	0.195 (6)	0.101 (4)	0.076 (3)	0.032 (4)	0.004 (4)	0.017 (3)
N1	0.0516 (14)	0.0506 (15)	0.0675 (17)	0.0005 (11)	0.0100 (13)	−0.0047 (12)
N2	0.0477 (14)	0.0553 (15)	0.0660 (17)	−0.0051 (11)	0.0173 (12)	−0.0059 (12)
C1	0.0376 (14)	0.0565 (18)	0.060 (2)	−0.0061 (12)	0.0143 (13)	−0.0038 (15)
C2	0.0357 (14)	0.0509 (17)	0.0553 (18)	−0.0017 (12)	0.0121 (12)	−0.0022 (13)
C3	0.0452 (15)	0.063 (2)	0.0500 (17)	−0.0050 (13)	0.0085 (13)	−0.0020 (14)
C4	0.0589 (18)	0.0581 (19)	0.059 (2)	0.0031 (14)	0.0046 (15)	0.0098 (15)
C5	0.0559 (18)	0.0522 (18)	0.063 (2)	0.0081 (13)	0.0087 (15)	−0.0016 (15)
C6	0.0432 (15)	0.0542 (18)	0.0518 (17)	0.0053 (13)	0.0074 (13)	−0.0008 (14)
C7	0.0447 (15)	0.0525 (17)	0.0504 (18)	0.0053 (12)	0.0085 (13)	0.0065 (13)
C8	0.0573 (18)	0.0546 (18)	0.0519 (18)	0.0078 (14)	0.0069 (14)	−0.0023 (13)
C9	0.071 (2)	0.111 (3)	0.069 (2)	0.020 (2)	−0.0006 (18)	−0.007 (2)
C10	0.0654 (19)	0.0571 (18)	0.0490 (17)	0.0085 (15)	0.0102 (15)	−0.0058 (14)
C11	0.092 (3)	0.112 (3)	0.054 (2)	0.021 (2)	0.0129 (18)	−0.0125 (19)
C12	0.0551 (17)	0.0531 (18)	0.0540 (18)	0.0050 (14)	0.0212 (14)	−0.0063 (14)
C13	0.072 (2)	0.056 (2)	0.078 (2)	0.0121 (16)	0.0171 (18)	−0.0117 (16)
C14	0.073 (2)	0.054 (2)	0.086 (2)	−0.0048 (16)	0.0092 (19)	−0.0178 (17)
C15	0.0517 (17)	0.061 (2)	0.0591 (19)	−0.0024 (14)	0.0079 (14)	−0.0079 (15)
C16	0.0511 (16)	0.0510 (17)	0.0499 (17)	−0.0044 (13)	0.0203 (13)	−0.0050 (13)
C17	0.0474 (17)	0.0536 (18)	0.0587 (19)	−0.0001 (14)	0.0242 (14)	−0.0004 (14)
C18	0.0505 (16)	0.0525 (18)	0.0560 (17)	−0.0027 (13)	0.0156 (14)	−0.0093 (13)
C19	0.067 (2)	0.071 (2)	0.079 (3)	0.0006 (16)	0.0034 (19)	−0.0085 (18)
C20	0.076 (3)	0.082 (3)	0.089 (3)	−0.0013 (19)	−0.011 (2)	−0.004 (2)
C21	0.058 (2)	0.087 (3)	0.123 (4)	−0.0026 (19)	−0.011 (2)	0.010 (2)
C22	0.0490 (18)	0.068 (2)	0.110 (3)	0.0052 (16)	0.0142 (19)	0.009 (2)
C23	0.0467 (16)	0.0398 (15)	0.083 (2)	0.0018 (12)	0.0147 (16)	0.0018 (15)
C24	0.0496 (16)	0.0379 (16)	0.077 (2)	0.0009 (12)	0.0226 (15)	0.0019 (13)
C25	0.063 (2)	0.0564 (19)	0.087 (3)	0.0040 (15)	0.0340 (19)	0.0061 (17)
C26	0.090 (3)	0.067 (2)	0.090 (3)	0.0024 (19)	0.052 (2)	0.0011 (19)
C27	0.089 (3)	0.080 (2)	0.068 (2)	−0.013 (2)	0.030 (2)	−0.0066 (18)
C28	0.063 (2)	0.075 (2)	0.073 (2)	−0.0135 (16)	0.0221 (18)	−0.0075 (18)

### Geometric parameters (Å, °)

**Table:**

Co1—N2	2.079 (2)	C10—C12	1.492 (4)
Co1—O4^i^	2.088 (2)	C10—C11	1.515 (4)
Co1—O2	2.091 (2)	C11—H11A	0.9600
Co1—N1	2.099 (2)	C11—H11B	0.9600
Co1—O3^i^	2.1602 (18)	C11—H11C	0.9600
Co1—O1	2.2030 (19)	C12—C13	1.385 (4)
O1—C1	1.257 (3)	C12—C18	1.385 (4)
O2—C1	1.262 (3)	C13—C14	1.381 (4)
O3—C17	1.264 (3)	C13—H13	0.9300
O3—Co1^ii^	2.1602 (18)	C14—C15	1.369 (4)
O4—C17	1.254 (3)	C14—H14	0.9300
O4—Co1^ii^	2.088 (2)	C15—C16	1.381 (3)
O1W—H11	0.8400	C15—H15	0.9300
O1W—H12	0.8401	C16—C18	1.383 (4)
N1—C19	1.330 (4)	C16—C17	1.487 (4)
N1—C23	1.344 (4)	C17—Co1^ii^	2.451 (3)
N2—C28	1.330 (3)	C18—H18	0.9300
N2—C24	1.345 (3)	C19—C20	1.372 (4)
C1—C2	1.483 (4)	C19—H19	0.9300
C2—C7	1.387 (3)	C20—C21	1.366 (5)
C2—C3	1.390 (4)	C20—H20	0.9300
C3—C4	1.371 (4)	C21—C22	1.367 (5)
C3—H3	0.9300	C21—H21	0.9300
C4—C5	1.382 (4)	C22—C23	1.395 (4)
C4—H4	0.9300	C22—H22	0.9300
C5—C6	1.384 (4)	C23—C24	1.473 (4)
C5—H5	0.9300	C24—C25	1.384 (4)
C6—C7	1.385 (4)	C25—C26	1.366 (4)
C6—C8	1.501 (4)	C25—H25	0.9300
C7—H7	0.9300	C26—C27	1.387 (5)
C8—C10	1.325 (4)	C26—H26	0.9300
C8—C9	1.510 (4)	C27—C28	1.375 (4)
C9—H9A	0.9600	C27—H27	0.9300
C9—H9B	0.9600	C28—H28	0.9300
C9—H9C	0.9600		
			
N2—Co1—O4^i^	96.57 (8)	C10—C11—H11B	109.5
N2—Co1—O2	99.46 (8)	H11A—C11—H11B	109.5
O4^i^—Co1—O2	156.66 (8)	C10—C11—H11C	109.5
N2—Co1—N1	77.60 (10)	H11A—C11—H11C	109.5
O4^i^—Co1—N1	95.09 (8)	H11B—C11—H11C	109.5
O2—Co1—N1	104.82 (8)	C13—C12—C18	117.3 (3)
N2—Co1—O3^i^	95.36 (9)	C13—C12—C10	123.2 (3)
O4^i^—Co1—O3^i^	61.75 (7)	C18—C12—C10	119.5 (3)
O2—Co1—O3^i^	99.77 (8)	C12—C13—C14	120.9 (3)
N1—Co1—O3^i^	155.20 (8)	C12—C13—H13	119.5
N2—Co1—O1	155.19 (8)	C14—C13—H13	119.5
O4^i^—Co1—O1	106.98 (8)	C15—C14—C13	120.8 (3)
O2—Co1—O1	60.85 (7)	C15—C14—H14	119.6
N1—Co1—O1	92.34 (9)	C13—C14—H14	119.6
O3^i^—Co1—O1	102.51 (8)	C14—C15—C16	119.7 (3)
C1—O1—Co1	87.29 (16)	C14—C15—H15	120.1
C1—O2—Co1	92.23 (17)	C16—C15—H15	120.1
C17—O3—Co1^ii^	87.33 (16)	C15—C16—C18	119.0 (3)
C17—O4—Co1^ii^	90.83 (16)	C15—C16—C17	120.8 (3)
H11—O1W—H12	108.6	C18—C16—C17	120.2 (3)
C19—N1—C23	118.5 (3)	O4—C17—O3	120.0 (3)
C19—N1—Co1	125.8 (2)	O4—C17—C16	120.2 (3)
C23—N1—Co1	115.7 (2)	O3—C17—C16	119.8 (3)
C28—N2—C24	119.2 (3)	O4—C17—Co1^ii^	58.40 (14)
C28—N2—Co1	124.42 (19)	O3—C17—Co1^ii^	61.68 (14)
C24—N2—Co1	116.3 (2)	C16—C17—Co1^ii^	177.01 (19)
O1—C1—O2	119.5 (3)	C16—C18—C12	122.3 (3)
O1—C1—C2	121.0 (3)	C16—C18—H18	118.9
O2—C1—C2	119.4 (3)	C12—C18—H18	118.9
C7—C2—C3	118.8 (3)	N1—C19—C20	123.1 (4)
C7—C2—C1	120.5 (3)	N1—C19—H19	118.5
C3—C2—C1	120.6 (2)	C20—C19—H19	118.5
C4—C3—C2	120.0 (3)	C21—C20—C19	118.7 (4)
C4—C3—H3	120.0	C21—C20—H20	120.6
C2—C3—H3	120.0	C19—C20—H20	120.6
C3—C4—C5	120.3 (3)	C22—C21—C20	119.4 (3)
C3—C4—H4	119.9	C22—C21—H21	120.3
C5—C4—H4	119.9	C20—C21—H21	120.3
C4—C5—C6	121.2 (3)	C21—C22—C23	119.3 (3)
C4—C5—H5	119.4	C21—C22—H22	120.3
C6—C5—H5	119.4	C23—C22—H22	120.3
C5—C6—C7	117.7 (3)	N1—C23—C22	120.9 (3)
C5—C6—C8	122.2 (3)	N1—C23—C24	115.2 (2)
C7—C6—C8	120.0 (2)	C22—C23—C24	123.9 (3)
C6—C7—C2	122.0 (3)	N2—C24—C25	121.1 (3)
C6—C7—H7	119.0	N2—C24—C23	115.2 (2)
C2—C7—H7	119.0	C25—C24—C23	123.7 (3)
C10—C8—C6	121.8 (3)	C26—C25—C24	119.1 (3)
C10—C8—C9	124.6 (3)	C26—C25—H25	120.5
C6—C8—C9	113.6 (2)	C24—C25—H25	120.5
C8—C9—H9A	109.5	C25—C26—C27	120.1 (3)
C8—C9—H9B	109.5	C25—C26—H26	119.9
H9A—C9—H9B	109.5	C27—C26—H26	119.9
C8—C9—H9C	109.5	C28—C27—C26	117.6 (3)
H9A—C9—H9C	109.5	C28—C27—H27	121.2
H9B—C9—H9C	109.5	C26—C27—H27	121.2
C8—C10—C12	122.1 (2)	N2—C28—C27	122.9 (3)
C8—C10—C11	123.8 (3)	N2—C28—H28	118.6
C12—C10—C11	114.1 (3)	C27—C28—H28	118.6
C10—C11—H11A	109.5		
			
N2—Co1—O1—C1	38.9 (3)	C6—C8—C10—C12	−1.7 (4)
O4^i^—Co1—O1—C1	−160.09 (16)	C9—C8—C10—C12	177.1 (3)
O2—Co1—O1—C1	−1.82 (15)	C6—C8—C10—C11	179.9 (3)
N1—Co1—O1—C1	103.86 (16)	C9—C8—C10—C11	−1.3 (5)
O3^i^—Co1—O1—C1	−96.14 (16)	C8—C10—C12—C13	72.2 (4)
N2—Co1—O2—C1	−162.06 (17)	C11—C10—C12—C13	−109.3 (3)
O4^i^—Co1—O2—C1	65.2 (3)	C8—C10—C12—C18	−109.3 (3)
N1—Co1—O2—C1	−82.50 (18)	C11—C10—C12—C18	69.2 (4)
O3^i^—Co1—O2—C1	100.77 (17)	C18—C12—C13—C14	0.4 (4)
O1—Co1—O2—C1	1.81 (15)	C10—C12—C13—C14	178.9 (3)
N2—Co1—N1—C19	−179.5 (2)	C12—C13—C14—C15	1.1 (5)
O4^i^—Co1—N1—C19	−83.9 (2)	C13—C14—C15—C16	−1.1 (5)
O2—Co1—N1—C19	83.8 (2)	C14—C15—C16—C18	−0.3 (4)
O3^i^—Co1—N1—C19	−103.9 (3)	C14—C15—C16—C17	−179.5 (3)
O1—Co1—N1—C19	23.4 (2)	Co1^ii^—O4—C17—O3	−2.0 (3)
N2—Co1—N1—C23	−1.95 (18)	Co1^ii^—O4—C17—C16	176.9 (2)
O4^i^—Co1—N1—C23	93.68 (19)	Co1^ii^—O3—C17—O4	2.0 (3)
O2—Co1—N1—C23	−98.61 (19)	Co1^ii^—O3—C17—C16	−177.0 (2)
O3^i^—Co1—N1—C23	73.7 (3)	C15—C16—C17—O4	−3.1 (4)
O1—Co1—N1—C23	−159.04 (19)	C18—C16—C17—O4	177.8 (3)
O4^i^—Co1—N2—C28	86.1 (2)	C15—C16—C17—O3	175.9 (2)
O2—Co1—N2—C28	−76.9 (2)	C18—C16—C17—O3	−3.3 (4)
N1—Co1—N2—C28	179.9 (2)	C15—C16—C18—C12	1.8 (4)
O3^i^—Co1—N2—C28	24.0 (2)	C17—C16—C18—C12	−179.0 (2)
O1—Co1—N2—C28	−112.2 (3)	C13—C12—C18—C16	−1.8 (4)
O4^i^—Co1—N2—C24	−91.99 (19)	C10—C12—C18—C16	179.6 (3)
O2—Co1—N2—C24	105.04 (19)	C23—N1—C19—C20	0.8 (4)
N1—Co1—N2—C24	1.81 (18)	Co1—N1—C19—C20	178.3 (2)
O3^i^—Co1—N2—C24	−154.10 (19)	N1—C19—C20—C21	−1.0 (5)
O1—Co1—N2—C24	69.7 (3)	C19—C20—C21—C22	0.5 (5)
Co1—O1—C1—O2	3.0 (3)	C20—C21—C22—C23	0.2 (5)
Co1—O1—C1—C2	−175.3 (2)	C19—N1—C23—C22	−0.1 (4)
Co1—O2—C1—O1	−3.2 (3)	Co1—N1—C23—C22	−177.9 (2)
Co1—O2—C1—C2	175.1 (2)	C19—N1—C23—C24	179.6 (2)
O1—C1—C2—C7	5.8 (4)	Co1—N1—C23—C24	1.8 (3)
O2—C1—C2—C7	−172.5 (2)	C21—C22—C23—N1	−0.4 (4)
O1—C1—C2—C3	−175.7 (2)	C21—C22—C23—C24	180.0 (3)
O2—C1—C2—C3	6.0 (4)	C28—N2—C24—C25	1.0 (4)
C7—C2—C3—C4	−1.3 (4)	Co1—N2—C24—C25	179.2 (2)
C1—C2—C3—C4	−179.8 (2)	C28—N2—C24—C23	−179.6 (2)
C2—C3—C4—C5	1.0 (4)	Co1—N2—C24—C23	−1.4 (3)
C3—C4—C5—C6	0.2 (4)	N1—C23—C24—N2	−0.3 (3)
C4—C5—C6—C7	−1.1 (4)	C22—C23—C24—N2	179.4 (3)
C4—C5—C6—C8	174.7 (3)	N1—C23—C24—C25	179.1 (3)
C5—C6—C7—C2	0.8 (4)	C22—C23—C24—C25	−1.2 (4)
C8—C6—C7—C2	−175.1 (2)	N2—C24—C25—C26	−2.5 (4)
C3—C2—C7—C6	0.4 (4)	C23—C24—C25—C26	178.2 (3)
C1—C2—C7—C6	178.9 (2)	C24—C25—C26—C27	1.5 (5)
C5—C6—C8—C10	74.1 (4)	C25—C26—C27—C28	0.8 (5)
C7—C6—C8—C10	−110.3 (3)	C24—N2—C28—C27	1.5 (4)
C5—C6—C8—C9	−104.9 (3)	Co1—N2—C28—C27	−176.5 (2)
C7—C6—C8—C9	70.8 (3)	C26—C27—C28—N2	−2.4 (5)

Symmetry codes: (i) −*x*+1/2, *y*−1/2, −*z*+3/2; (ii) −*x*+1/2, *y*+1/2, −*z*+3/2.

### Hydrogen-bond geometry (Å, °)

**Table:**

*D*—H···*A*	*D*—H	H···*A*	*D*···*A*	*D*—H···*A*
O1w—H11···O1	0.84	1.98	2.812 (5)	173
